# Single Molecule Analysis Research Tool (SMART): An Integrated Approach for Analyzing Single Molecule Data

**DOI:** 10.1371/journal.pone.0030024

**Published:** 2012-02-20

**Authors:** Max Greenfeld, Dmitri S. Pavlichin, Hideo Mabuchi, Daniel Herschlag

**Affiliations:** 1 Department of Chemical Engineering, Stanford University, Stanford, California, United States of America; 2 Department of Biochemistry, Stanford University, Stanford, California, United States of America; 3 Department of Physics, Stanford University, Stanford, California, United States of America; 4 Department Applied Physics, Stanford University, Stanford, California, United States of America; Swiss Federal Institute of Technology Zurich, Switzerland

## Abstract

Single molecule studies have expanded rapidly over the past decade and have the ability to provide an unprecedented level of understanding of biological systems. A common challenge upon introduction of novel, data-rich approaches is the management, processing, and analysis of the complex data sets that are generated. We provide a standardized approach for analyzing these data in the freely available software package SMART: Single Molecule Analysis Research Tool. SMART provides a format for organizing and easily accessing single molecule data, a general hidden Markov modeling algorithm for fitting an array of possible models specified by the user, a standardized data structure and graphical user interfaces to streamline the analysis and visualization of data. This approach guides experimental design, facilitating acquisition of the maximal information from single molecule experiments. SMART also provides a standardized format to allow dissemination of single molecule data and transparency in the analysis of reported data.

## Introduction

The goal of traditional thermodynamic and kinetic methods has been to measure properties of ensembles and infer the behavior of individual molecules. Single molecule approaches provide a unique ability to directly visualize processes carried out by individual molecules and complexes [1], [2], [3], [4], [5], [6], [7], [8].

There is a rich history of single molecule approaches that have dominated mechanistic investigation of ion channels [9], [10]. More recently, fluorescence and force measurements at the single molecule level have greatly expanded the types of biological systems amenable to single molecule investigation [11], [12]. These studies have allowed the identification and study of rare states and events that would be difficult or impossible to infer from bulk studies and have revealed a remarkable extent of molecular heterogeneity that had not been apparent from bulk studies [13], [14], [15], [16], [17], [18].

Standardization is a ubiquitous challenge that must be faced when novel methods are introduced and widely adopted. Additionally, the dissemination of the actual experimental results is difficult when there are large datasets. This challenge was extensively discussed, and largely surmounted, for X-ray structural, microarray, and other genomic data [19], [20], [21], [22]. Single molecule experiments contain orders of magnitude more information than data from traditional bulk methods and are analyzed differently in different laboratories [23], [24], [25], [26], [27]. As a result, it is typically not possible to directly evaluate or reanalyze published conclusions from single molecule experiments.

The rapid growth of single molecule publications highlights the current need for standardization. At present, individual investigators have a host of data analysis strategies to choose from, as single molecule data is generated from many sources and its analysis has been subjected to extensive study. Nevertheless, the particular strategy implemented to analyze a particular single molecule experiment is usually lab-generated. While this approach can be most efficient for the individual who must balance the demands of data collection, analysis, and dissemination, it is inefficient in the longer term and for the broader community. A larger time investment by a subset of investigators to create a more general and efficient tool could result in enormous aggregate time savings. Such a tool could facilitate the rapid evaluation of experimental results, the comparison of results from different labs, and the reanalysis and reevaluation of published results in light of new data and models.

To meet these important challenges, we have developed the software package SMART: Single Molecule Analysis Research Tool. This package is freely available, easily implemented, and provides an integrated and convenient tool for data processing, analysis, and visualization.

## Results

SMART provides a means to rapidly organize and analyze the large and complex data sets generated in single molecule experiments. Ultimately, the researcher would like to use the single molecule data to build kinetic and thermodynamic models that account for the raw data and explain the behavior of the molecule(s) of interest. The process of analyzing single molecule data can be cumbersome, but, even more fundamentally, it is often difficult to relate errors and uncertainties from the raw data –traces for individual molecules– to uncertainties in the models obtained. This difficulty is exacerbated by the inherent stochasticity of single molecule measurements and the typically limited time window for data collection.

**Figure 1:**
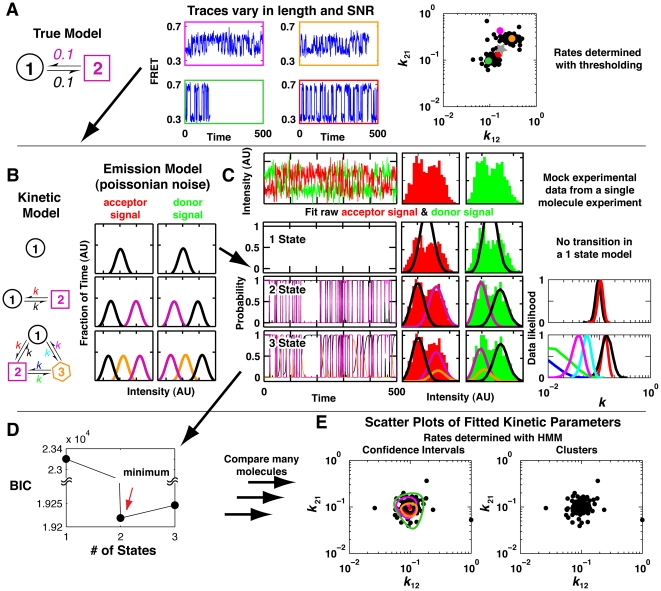
Workflow for SMART analysis of single molecule data. In a typical analysis of single molecule data the distribution of rate constants determined from a simple two-state system can appear heterogeneous because of uncertainties that arise from variation in trace length and SNR. SMART addresses these limitations. Part A (left) shows a simple model used to generate 200 simulated traces, with SNR's of either 4 or 12 and trace lengths determined by a photobleaching model (see Methods). Four representative traces are shown, and the time constants for these four molecules (colors) and each of the other simulated molecules (black) determined by threshold analysis are plotted on the right. The gray star represents the inferred transition rate assuming all the molecules arose from the same population of molecules. Panels B–E show analysis of the single molecule FRET data from Panel A subject to SMART analysis. The analysis is shown for one molecule, and the data for all molecules are compared in Panel E. (B) The user specifies a set of kinetic and emission models to be fit to the observed trace. (C) Traces are analyzed individually. The donor (green) and acceptor (red) intensities are plotted as a function of time and are used directly in the fits. The cumulative histograms for the intensity of each are plotted on the right and are fit during the analysis. Fits of the models to the data are shown for the one-, two-, and three-state models of Panel B. State occupancy probabilities are shown on the left, fitted emission distributions are depicted in the middle, and the inferred transition rates between states (*k*_xy_), and normalized likelihood values (confidence intervals) are plotted on the right (colors depict rate constants for different transitions). SMART is able to calculate confidence intervals for each of the fitted parameters. (D) The Bayesian information criterion (BIC) is used to select a model that best balances goodness of fit and the number of free parameters. The fit with the lowest Bayesian information criteria has the optimal fit. (E) Summary of data from steps (B) and (C) for the entire population of 200 molecules. The plots show different representations of uncertainties, with confidence intervals on the left (shown explicitly only for the colored traces from Panel A) and as clusters on the right (one cluster is shown). The molecules that segregated into two apparent classes by thresholding have overlapping confidence intervals (left) and fall in the same cluster (right) and thus do not provide evidence for distinct populations of molecules.

To underscore this point, Fig. 1A provides an example in which not accounting for the noise inherent to the measurements could lead an investigator to draw incorrect conclusions. Two hundred traces were generated from a simulation with a stochastic two-state model (*k*_12_ = *k*_21_ = 0.1), shown on the left of Fig. 1A, with individual molecules having a signal to noise ratio (SNR) of either 4 or 12 (See Methods); traces for four of these molecules are shown in the center of Fig. 1A. [*Note: Kinetic models are in discrete time throughout this paper. In this situation, the kinetic model parameters are transition probabilities per time step that take values between 0 and 1. In an experiment the time step is set by the sampling rate. Transition probabilities can be converted to rate constants approximately by multiplication by the sampling rate, or by the more accurate relationship given by equation S26 of Appendix S1.*] The traces were analyzed using the common thresholding analysis approach. Details of thresholding and alternative approaches are described in a later section. Kinetic data obtained from these molecules are plotted on the right, with the colored traces in the center represented by the same colors and additional black points for analogous simulated traces that are not shown. The analysis reveals two distinct clusters of molecules. However, the model underlying this simulation was a simple two-state model, with uniform values for the rate constants for all of the individual molecules.

The origin of this clustering is that molecules with lower SNR appear to have more transitions and thus give larger calculated rate constants. Thus, an investigator using threshold analysis could have concluded that the underlying molecular behavior was more complex than it actually is, with two types of molecules in the population that are kinetically distinct. If traces in the same experiment yield different thermodynamic or kinetic behaviors, the molecules and their behaviors are described as heterogeneous [13], [28], [29], [30], [31], [32]. A major focus of current single molecule experiments is to understand the origins of potential underlying heterogeneity. A standardized approach for evaluating the presence or absence of heterogeneity, which in this instance could prevent an erroneous conclusion, is needed.

A common approach for dealing with the limited amount of information contained in any single data trace is to make an assumption that all of the molecules follow a common kinetic model [25], [33], [34] –i.e., that there is no significant heterogeneity in the single molecule behavior. Rate constants determined by this approach of combining the data for the individual molecules are shown by the gray star in Fig. 1A (right); this approach, in some instances, does not yield a good approximation of the true rate constants; here the values differ by two-fold from the actual values.

To accurately determine the rate constants for the data in Fig. 1A and, more generally, to faithfully obtain models for each molecule –and avoid assuming a common model for all molecules, assignment of statistical confidence intervals for each molecule is needed. Statistical analysis is needed to determine if different molecules follow the same or different kinetic models and, if the same model is followed, to determine if the individual rate and equilibrium constants for different individual molecules are the same, within error, or different. The uncertainty for individual molecules will be different, depending on the length of the trace, the SNR of the trace, and the number of transitions that occur in the trace. Thus, each molecule must be analyzed independently. Indeed, the thresholding approach eliminates experimental information needed for statistical analysis, as it makes an absolute and local judgment as to what state a molecule is in and whether or not a transition has occurred, whereas in reality these judgments can only be made at a particular level of confidence –a level that depends on the aforementioned properties of the data for the molecule undergoing analysis.

**Figure 2:**
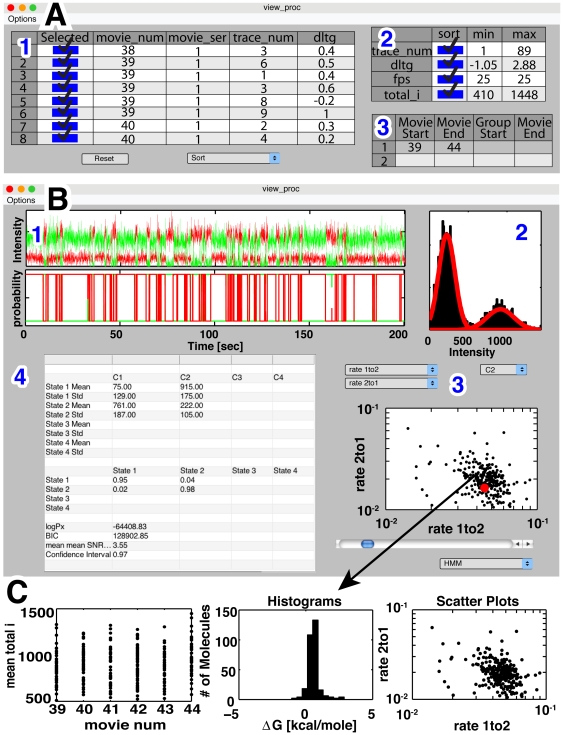
Highlights of SMART, an integrated data analysis tool that combines general HMM algorithms with graphical user interfaces to allow data to be visualized and rapidly analyzed. (A) Molecules can be selected on the basis of experiment type and/or fitted parameters: (A1) Fitted parameters can be inspected and molecules manually selected in tabular form. (A2) Molecules can be selected based on a user-specified range of experimental or fit values. (A3) Molecules can be selected by a user-defined experiment number or numbers. (B) Interactive data viewing environment allows inspection and plotting of raw data and fitted model parameters: (B1) A raw trace and the estimated state occupancies. (B2) Cumulative emission distributions and fitted emissions model. (User chooses which channel is shown.) (B3) Scatter plot of all molecules of the user-specified group. The red dot indicates the molecule that is summarized in B1, B2, and B4. (B4) Fitted model parameters for the indicated molecule displayed in tabular form. (C) The environment (B3) allows the rapid generation of data summaries for the specified molecules and displays them graphically; three additional data summary graph formats are shown in Part C.

**Figure 3:**
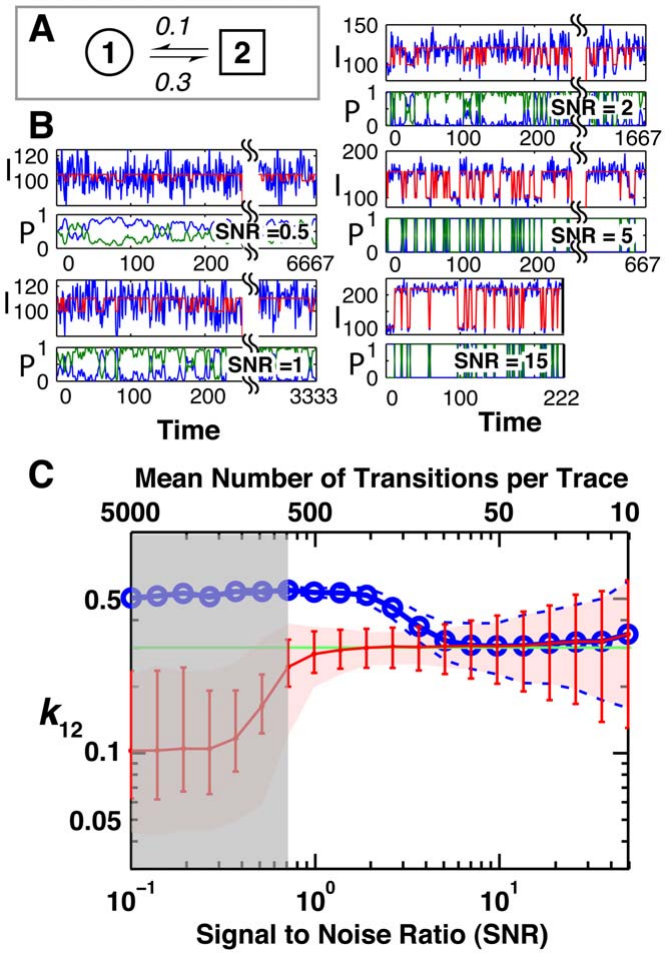
Comparison of HMM and thresholding for identifying the true rate constants from traces varying over a range of SNRs with trace length (not shown) inversely proportional to SNR to account for photobleaching in smFRET experiments. (A) The two-state kinetic model used in simulating traces over a range of SNRs. (B) Anecdotal traces at five different SNRs, simulated emissions (see Methods) are shown in blue and the true state being occupied is shown in red. Two-state HMM fits are shown below the simulated traces. The blue line indicates the probability of being in state 1 (low intensity) the green line indicates the probability of being in state 2 (high intensity). I and P on the ordinate of the traces indicate intensity and probability, respectively, for each SNR. (C) The average inferred rate constants obtained using thresholding (blue) and HMM modeling (red) as a function of the SNR. The true value, represented by the horizontal green line, is 0.3 (Panel A). The dotted blue line and red swath represent the region that bounds 90% of the determined rate constants from the 500 simulated traces analyzed for HMM and threshold fits, respectively. The mean number of transitions per trace is indicated at the top of the graph. As the difference in signal means for true transitions becomes negligible relative to the noise, the BIC indicates that a one-state fit provides the best fit to the data; this region is shown by the gray swath.

**Figure 4:**
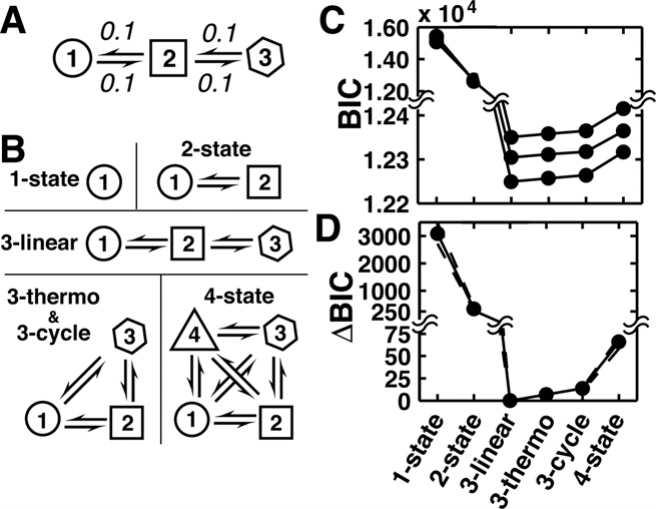
Testing the ability of the BIC to identify the true model. (A) Three-state model used to generate mock traces. In this model, states 1 and 2 had emission properties identical to the states in Fig. 3A (also see Methods), and the equivalent of the SNR of 4 from that figure was used. State 3 was added with emission halfway between these states, resulting in an effective SNR between states of 2. (B) Simulated traces were fit to six different HMM models. The 3-thermo and 3-cycle models have identical topology but 3-thermo was fit using a constraint of thermodynamic closure (i.e., the rate constants determined will satisfy detailed balance) and therefore has one fewer fitted parameter than 3-cycle. (C) Plots of the BIC for the six different models. Three BICs for three example traces are shown in black. (D) Same data as in part C except that the difference between the 3-linear BIC (lowest in all cases) and the BIC for the other models is plotted. The solid black line indicates the mean of this difference for 1000 traces and the dashed lines indicate 90% confidence intervals.

**Figure 5:**
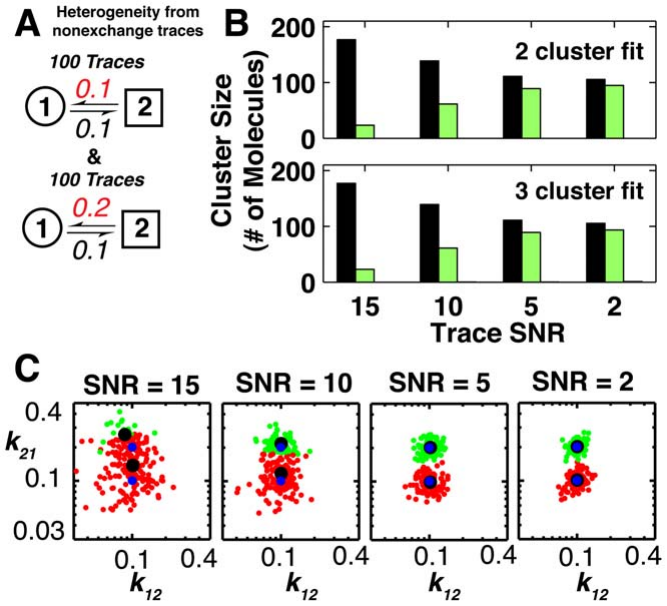
Clustering algorithms to identify two non-exchanging populations of molecules. (A) Traces with SNRs of 2, 5, 10 and 15 were generated from two non-exchanging pools of molecules (100 traces each) with one transition rate differing by two-fold. The traces were fit to two-state HMM models and subjected to clustering analysis in SMART. (B) Traces were fit with 1 to 4 clusters; the cluster size of the 2 and 3 cluster fits are shown while the 1 and 4 cluster fits are shown in Appendix S1. The black and green bars correspond to an individual cluster size at the indicated SNR; the bars corresponding to the third cluster in the third cluster fit is not visible due to its small size. (C) Scatter plots for two-cluster fits of the inferred rate constants. Black dots indicate the two inferred cluster centers, and blue dots indicate the true population centers.

**Figure 6:**
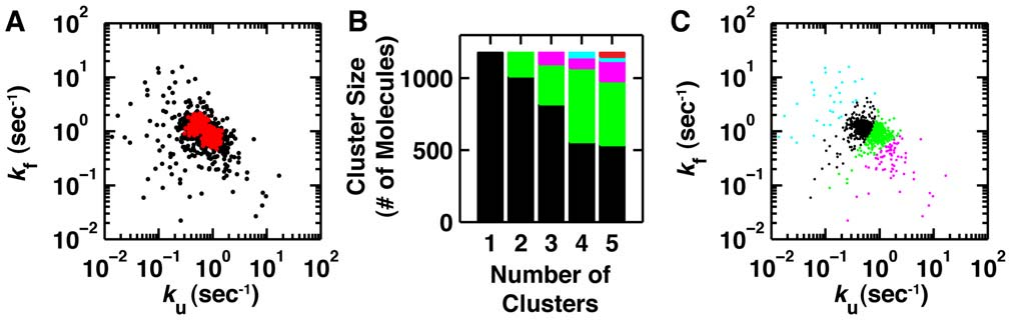
Analysis of heterogeneity in smP4–P6 RNA with simulations and SMART clustering. The smP4–P6 data and simulation analysis are from Greenfeld *et al.* [65]. (A) Folding and unfolding rates of smP4–P6 (black) were analyzed by simulating two non-exchanging populations of molecules whose rate constants differ by two-fold (red). By this analysis 80% of the molecules are accounted for by the simulated data. (B) The inferred cluster size for fits of 1 to 5 clusters of the folding and unfolding rates of smP4–P6. (C) Color-coded smP4–P6 kinetics from the four-cluster fit. The two central clusters account for 90% of the molecules.

SMART implements a workflow with tools that overcome the statistical limitations introduced in Fig. 1A and several other challenges associated with analyzing single molecule data. The features of SMART include a graphical interface that makes it easy to inspect and compare raw and processed data, algorithms for fitting data to a series of possible models that allow the goodness of fit to be assessed, clustering algorithms for grouping molecules based on the similarity of inferred parameters, and a data format that simplifies the sharing of raw and processed data. We begin by providing a general overview of the fitting procedures used in SMART (Fig. 1B–E), and we then describe the user interface (Fig. 2). Subsequent sections (Fig. 3, 4, 5, 6) provide a more in-depth explanation of key features introduced in Fig. 1B, C, D, E.

### Overview of the SMART Workflow

Single molecule traces can be generated from many different types of experimental techniques investigating diverse biological or biochemical systems. A trace typically consists of one or multiple signals, which have time-dependent intensity fluctuations that depend both on the dynamics of the system being studied and noise that is intrinsic to measurements with single molecule sensitivity. A key challenge in the data analysis is to identify with statistical rigor levels of discrete, stable intensity and transitions between those levels. We develop SMART to meet this challenge and to provide a convenient platform for analysis and sharing of data. The SMART workflow is schematized in Fig. 1B, C, D, E. Following this workflow should allow a user to develop thermodynamic and kinetic models and describe the level of confidence in such models.

In the first step (Fig. 1B) multiple user-specified kinetic models are put forward for analysis. Kinetic models can have an arbitrary number of states (circles, squares and hexagons) and transitions can be specified to occur between any states (arrows). Transitions between states are stochastic and the transition rate constants are constant in time, leading to dwell times spent in each state that are exponentially distributed, as is standard for rate processes. An observed signal with a particular signal intensity can correspond to one state or multiple states in the model. In addition to the kinetic model, a model of Poissonian or Gaussian noise is assumed for the signal observed in an experiment for each state in each channel (Fig. 1C, intensity histograms). Ultimately the noise model used depends on the details of the system being investigated, and a different noise model can be assumed for each input channel being fit. Thus, instead of calculating FRET, the observed signals in each channel (e.g., donor and acceptor) are fit directly. Direct fitting avoids unnecessary pre-processing of data and allows noise in each of the signals to be appropriately accounted for. After the data are fit to each of the specified models, the optimal model is selected.

The data are fit to Hidden Markov Models (HMM) (Fig. 1C), which are often used to analyze single molecule data as they are well-suited for data of this type [35], [36], [37], [38], [39], [40], [41], [42], [43]. Traces have stochastic variations from two sources –noise and actual transitions between states. HMMs jointly fit a combined kinetic and noise model to each individual trace. Parameters for the models (e.g., rate constants and the mean and standard deviation for the Gaussian or Poissonian noise) are determined for each molecule using maximum likelihood estimation (MLE) [44], [45]. For each state in the model we can determine the state probability at each time point in the trace (Fig. 1C, left); when a state probability is close to one, there is a high probability that the state is occupied. Fits of the noise model can be visualized by using the fitted parameters to generate curves that overlay with the cumulative intensity channels for each trace (Fig. 1C, middle).

Importantly, SMART also implements an algorithm for determining the confidence intervals for each of the parameters inferred in fitting an HMM to data. The likelihood-ratio test for HMM's was recently described by Giudici *et al.* [46], [47], but to the best of our knowledge has not seen use in the single molecule community. In our example (Fig. 1C, right), confidence intervals were determined for the kinetic parameters. Knowing the confidence with which a kinetic parameter has been determined is critical to assessing if molecules are behaving differently or if spread in the determined parameters is consistent with uncertainties in the measurement.

From the multiple kinetic models that are fit to the data, a model that optimally describes the data must be chosen. Higher-order models (i.e., those with more degrees of freedom) always fit data better than lower-order models. Thus, a statistical test is required to justify added degrees of freedom. As model parameters are determined using MLE, the Bayesian information criteria (BIC) is a natural metric for selecting the optimal model in SMART [45],[48],[49]. The BIC rewards models that fit the data well and punishes extra free parameters. The BIC is determined and compared for fits to different model types. The model with the lowest BIC is optimal in balancing goodness of fit with simplicity. This comparison can be made graphically by plotting the BIC as a function of the model complexity (Fig. 1D). The BIC has a rigorous theoretical basis, but has limitations that are discussed in a later section.

Once individual traces have been fit, the next step is to bring the information for all of the molecules together. A fundamental question is whether there is molecular heterogeneity in the experiment. To evaluate the presence of heterogeneity with SMART two tools are provided (Fig. 1E). First, scatter plots of fitted parameters and their corresponding confidence intervals can be used to assess by inspection if there is overlap in the confidence ranges for all, nearly all, or subsets of the molecules (Fig. 1E, left; confidence intervals shown only for the four molecules from Fig. 1A). Second, molecules can be clustered based on the similarity of the fitted model parameters (e.g., rate constants) and the uncertainties associated with them. Clustering provides a quantitative measure of the overlap between molecules [50]. However, clustering has not been widely used for evaluating the heterogeneity that is typically seen in single molecule experiments. In SMART, the optimal number of clusters used to describe the data is determined using a technique similar to the BIC model selection criterion used in Fig. 1D. For the SMART analysis of the simulated data in Fig. 1A, one cluster optimally fits the data (Fig. 1E, right), providing no indication of heterogeneity in agreement with the input model. Analogous exercises with distinct underlying populations reproduce the number and properties of these subpopulations (Fig. 5 below).

### SMART: The software package

For someone designing, optimizing, and conducting single molecule experiments, the data analysis steps outlined in Fig. 1B–E are carried out on a regular basis and can be enormously time-consuming. SMART is designed to make this analysis easy so that experimentalists can focus their attention on comparing and interpreting data and designing experiments that yield more information about the system being studied. SMART has a graphical interface that streamlines access to built-in functions and allows the rapid viewing of raw and processed data. Standardization of data processing and visualization has been important for genomics and macromolecular structure determination [19], [20], [21], [22]. It seems likely that standardization by SMART or a similar approach will have an analogous impact for those carrying out single molecule experiments. All of SMART and the interface is written in the widely used MATLAB™ programming language, allowing modification of SMART by advanced users and direct interfacing with MATLAB™'s extensive built-in functionalities. SMART is freely available and can be downloaded from the website simtk.org maintained by Simbios (the NIH Center for Physics-based Simulations of Biological Structures – simbios.stanford.edu) at Stanford University.

The SMART software package is extensive, and a full description of all functions is described in the user manual that is included with the package. Some key features of the interface are depicted in Fig. 2. After having fit individual molecules to kinetic models (interface not shown, see SMART user manual), groups of molecules can be selected for further analysis. Molecules can be selected by inspection (Fig. 2A.1), by specifying a range of desired fitted parameters (Fig. 2A.2), or by identifiers that specify the day and type of experiment that was completed (Fig. 2A.3). Once molecules are grouped, they are displayed in the interface depicted in Fig. 2B. This interface depicts the raw data and inferred state distributions for an individual molecule (Fig. 2B.1), fits to the noise model (Fig. 2B.2), and all of the fitted parameters in tabular form (Fig. 2B.4). Plots of fitted parameters can be generated (Fig. 2B.3) instantaneously for all molecules selected in Fig. 2A and easily exported (Fig. 2C) for future use. Three classes of plots can be generated: (1) histograms of a single parameter vs. the number of times it was observed; (2) plots of a single parameter in rank order from the lowest to highest observed value; and (3) scatter plots of two fitted parameters. Features of the SMART interface not shown include fitting of data to models, model selection, and clustering.

### SMART Uses General HMM Algorithms to Fit Models to Single Molecule Data

#### Comparison of HMM and thresholding

Single molecule experiments monitor the temporal changes of a molecule by recording one or multiple experimental observables, such as changes in donor or acceptor intensity in a FRET experiment or a distance fluctuations measurement. Typically these data are stereotyped by the presence of rapid (beyond the temporal resolution of an experiment) transitions between states of stable intensity. When the data have discrete hops (or can be well approximated by this form) identifying the occurrence of transitions in intensity levels is a key challenge of the analysis.

Two general means have been used to identify changes in intensity in single molecule data. Thresholding identifies states by defining intensity thresholds that stereotype each state [51]. This approach is intuitive and easiest to implement. However, fitting the data to a statistical model using Bayesian inference is a more general approach [45]. A large class of models that is fit using this approach are HMMs; as discussed below, HMMs make minimal assumptions about the underlying origins of the data signals [45]. In experimental systems where the data are not characterized by discrete hops (i.e., where continual variation in intensity levels is observed) or where a detailed understanding of the system can justify the creation of more complex models to fit to the data alternative analysis procedures can be used [52], [53], [54].

To compare the performance of thresholding and HMM in inferring rate constants, we simulated traces for a single fluorophore-labeled molecule fluctuating between two states with distinct levels of fluorescence, inferred rate constants by thresholding and by fitting to two-state HMMs, and compared the inferred values to the true rate constants. This and all subsequent simulations used Poissonian emission noise (Fig. 3A and Methods), as this is the theoretical noise model for a single fluorophore [55]. In these simulations the SNR and trace length were varied inversely to reflect faster photobleaching that occurs at higher SNR; the product of SNR and trace length were held constant to reflect a simple model of the dependence of the dye photobleaching time on laser intensity and thus SNR (see Methods). The effects of alternative photobleaching models on the determined rate constants are considered in Fig. S1 of Appendix S1, since a simple linear dependence is not always observed experimentally. Fig. 3B is shown to provide examples of the physical appearance of real single molecule data with typical SNR values used throughout this analysis (see Fig. S1 of Appendix S1). Traces are shown at five different SNRs (blue lines) with the state lifetimes (i.e., rate constants) fixed. The red lines show the true state that each molecule occupies at each time. As the lengths of the traces vary considerably (from about 200 to 30,000 time steps), a constant window of about 250 time steps is shown.

The thresholding procedure defines a value for a signal that, once crossed, indicates that a state transition has occurred. Histograms of the dwell times between transitions are then fit to kinetic models; in this instance a model of exponentially distributed dwell times was used. The blue line in Fig. 3C indicates the mean inferred *k*_12_ rate constants determined using thresholding for all of the simulated traces at a given SNR (only one of the determined rate constants is shown since they are both determined with comparable accuracy). The region bounded by the dashed blue lines indicates the spread in the inferred rate constants that accounts for 90% of all inferred values from fits to 500 traces (these can be well approximated with 100 traces but are less smooth).

Comparison of this analysis to the actual rate constant (*k*_12_ = 0.3) shows that thresholding works best at SNRs greater than ∼5 for the conditions simulated. Below this SNR false transitions produce an upward bias in the determined rate constants with completely erroneous values obtained at SNRs lower than 3 to 4. At low SNR, the rates converge to a value of 0.5; the value of 0.5 is expected for fitting noise because every time point has an equal probability of being above or below the threshold, so that there is a probability of an apparent transition at each timepoint of 0.5. At high SNR (i.e., short trace length), it is common to have traces with zero transitions. These traces only provide an upper bound on one of the state lifetimes and no information on the other. Therefore, traces with fewer than two transitions were excluded from this analysis, leading to an upward bias in the mean number of transitions per trace. Above a SNR of ∼30, a slight upward bias in the rate constants is observed. This feature becomes more pronounced for lower rates because even fewer transitions are present in the data (Fig. S2 of Appendix S1).

HMM fitting, as outlined in the next section, provides a good fit at lower SNRs compare to thresholding (Fig. 3C). This can be very important in single molecule fluorescence experiments as the use of lower SNRs typically makes it possible to achieve longer individual-molecule data traces, as a result of reduced photobleaching rate, and this in turn aids in the analysis of potential molecular heterogeneity (as will be discussed below). HMM performs better than thresholding because instead of determining the hidden state locally in time for each data point, it assigns a state occupation probability conditioned on all past and future observations and based on the noise model for the system. The power of the HMM method can be seen in the traces of Fig. 3B. At high SNR, the state probabilities are essentially 1 or 0, so that there is little difference from thresholding. However, at lower SNR, as is often required to obtain sufficiently long data traces in practice, the probabilistic state assignments from HMM differ substantially from the absolute threshold assignment. These simulations also help establish an optimal SNR regime for carrying out experiments.

#### Implementation of HMM in SMART

SMART determines kinetic parameters by fitting HMMs to the raw data. HMMs have been used for single molecule data analysis, and the fitting procedures used in SMART provide notable improvements over commonly available implementations.

HMMs are a flexible model type that have seen use in diverse fields such as voice recognition software and genomic sequence alignment [45]. HMMs are a more general form of Markov Models (MM). A MM is composed of directly observable states that are connected by stochastic transitions with rate constants that are constant in time, which is equivalent to having exponentially distributed dwell times in each state. A MM can have any number of states, and these states can be connected in any manner. MMs take as inputs long series of data (e.g., a string of characters or a time series of binary data), and multiple data sources can be fit in an MM. However, in single molecule measurements states cannot be directly observed and inferred with absolute confidence. The state that a molecule occupies is masked by noise, and it is also possible that multiple states produce identical signals. HMMs are ideal for fitting this type of data, as HMMs have all the properties of MM, but handles states that cannot be directly observed [45].

For each specified model, the HMM algorithms in SMART fit a statistical model to the data to determine the parameters that best fit the data and the confidence intervals associated with those parameters. The optimal model parameters for a model with K hidden states are determined to maximize the likelihood of the data given a model [p(data | model)] given by equation 1,
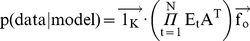
(1)where 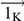 is the unit vector of length K, E_t_ is the emission probability matrix, A^T^ is the transition probability matrix, and 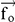 is the best guess of the initial state of the system. Equation 1 is the key equation evaluated in performing the HMM fit, and a detailed description of these parameters and this equation is given in Appendix S1. The MLE is determined using the computationally efficient Baum-Welch algorithm [44], [56]. A key feature of SMART is the determination of confidence intervals for the fitted HMM parameters. Once the MLE has been calculated, determining these confidence bounds for any inferred parameters is possible. This determination is done by varying the parameter value around the MLE and recording the decrease in the data likelihood. A confidence bound is determined by rejecting all models that produce the observed data with less than a specified threshold [46].

In Fig. 3C, the mean inferred rate constants determined using HMMs for all of the simulated traces at each SNR are indicated by the red line. The region bounded by the opaque red swath indicates the spread in the inferred rate constants that accounts for 90% of all inferred rate constants determined by fitting 500 traces. HMM fits are able to correctly infer rate constants at SNRs considerably lower than thresholding can, at SNRs of ∼1 in our example. Fig. S1 of Appendix S1 shows that the SNR cutoff can be lower for states with longer state lifetimes. The more reliable performance of HMMs arises from the fact that inferred state occupation probabilities are not sensitive to occasional jumps of the signal across a threshold. Nevertheless, at low SNR the fitted rate constants converge to a value of ∼0.1 (and not 0.5, as would an unbiased estimator). This result shows that HMM fits do not provide an unbiased estimator of model parameters and that the bias is larger in the low SNR regime. Fig. S2 of Appendix S1 shows, using two additional examples with different transition rates, that the inferred rate constants are accurately determined down to an SNR of ∼1 but also converge to a value of 0.1 at low SNRs. At high SNR the performance of HMM fits and thresholding are the same because the uncertainty in indentifying a state becomes small.

To test the accuracy of the calculated confidence values determined during HMM fitting we compared the calculated confidence bounds to an uncertainty measurement determined with simulation. The confidence bounds determined as part of the HMM fits for the rate constants are shown by the red vertical error bars in Fig. 3C. Comparison of this region to the 90% confidence intervals calculated from fits to 500 simulated traces (Fig. 3, opaque red region) indicates good agreement between the two measures of uncertainty. Computation of the confidence intervals relies on the assumption that the data likelihood is approximately Gaussian near the maximum likelihood estimator. This assumption can be invalid at low SNR when transitions are not reliably observed and at high SNR when transitions are reliably observed but few in number due to smaller observation times. For the high SNR case an investigator can assess this assumption for a particular data set by plotting the data likelihood to see if the distributions are Gaussian (e.g. as shown in Fig. 1C right). Below an SNR of 1, the confidence intervals calculated with SMART underestimate the true uncertainty in the inferred rate. This discrepancy can be reduced by accounting for correlated uncertainties in the fits, as discussed in Appendix S1 Fig. S3. Above a SNR of 3 these discrepancies are relatively small (with a mean discrepancy for SNRs between 1 and 50 of ∼10% overestimating the uncertainty in the inferred rate, reflecting the increasingly poor fit of a Gaussian to the data likelihood due to shorter observation times), making the confidence intervals a good estimator of the uncertainty in the inferred parameters over a wide range of SNRs. Evidence of the poor fit quality in the low SNR regime can be gained from the model selection criteria. In the case of Fig. 3C traces with an SNR below 0.7 are more appropriately fit with a one-state model rather than the two-state model; this indicates a lack of confidence in the fit and derived parameters for the two state model (see the next section for a further discussion).

The analysis of Fig. 3 shows there are regimes in which rate constants can be accurately determined. However, the ability to fit a trace depends both on the state lifetimes and the relationship between SNR and trace length. Fig. S1 of Appendix S1 systematically explores the quality of fits for a two-state system (*k*_12_ = *k_21_* = 0.1) as the relationship between SNR and trace length is systematically varied. State lifetime can have a similarly complex effect on the ability to infer rate constants. Indeed the SNR limit of 0.7 for obtaining accurate rate constants in Fig. 3C is only true for those simulated conditions. As demonstrated in Fig. S1 of Appendix S1, as lifetimes increase, accurate rate constants can be inferred from traces at significantly lower SNRs.

HMMs in general allow transitions between states to occur that violate microscopic reversibility and, as a result, fits to HMMs are not guaranteed to have thermodynamic closure (detailed balance) –i.e., to be thermodynamically correct. Many single molecule experiments, including those conducted on motor proteins, polymerases and helicases are not at thermodynamic equilibrium. However, imposing this constraint is appropriate when experiments are carried out at thermodynamic equilibrium. SMART overcomes this basic limitation by allowing HMMs to be fit using a constraint of thermodynamic closure. A detailed description of this fitting procedure is the subject of a theoretical study that will be published elsewhere, and a brief description is provided in Appendix S1.

A more general limitation of HMMs arises in that certain models are not accurately described or well approximated by HMMs with a few states. A common example in the biophysical literature is the stretched exponential model, which has received considerable attention as a possible description for the fast folding of proteins [57], [58], [59], [60] and is often used as a phenomenological model. Although an HMM with many states can approximate the form of a stretched exponential, this approximation leads to the fitting of many kinetic parameters in contrast to the two parameters in the analytical form of a stretched exponential and is therefore not ideal. However, for many systems a simple HMM with a limited number of states can rapidly provide good fits to the data, and predicted behaviors from the best fit model can be subjected to subsequent experimental tests. HMMs can also aid in experimental design as a researcher determines optimal conditions that account for the tradeoffs between SNR and trace lifetimes (Fig. 3C and Fig. S1 of Appendix S1).

### Determination of the Optimal Model

The flexibility of the HMM architecture enables many different types of models to be fit to data. In most cases an investigator is interested in identifying the simplest model (with the smallest number of free parameters) that fits the data well; this model will then be further tested and refined in subsequent studies as a kinetic, thermodynamic, and mechanistic understanding of the system is developed.

While model size estimation is not a solved problem in hidden Markov model inference, the Bayesian Information Criterion (BIC) is a widely adopted method with a theoretical basis [45], [48] and is computationally accessible [61], [62]. The BIC's performance as a function of data length and SNR is not completely understood and has only been investigated in a few cases [61], [63] and the method does not provide the user with a quantitative measure of confidence in its output. Nevertheless, there are no easily accessible methods that clearly outperform BIC. We have therefore implemented the BIC as a guide to the experimenter in assessing the dynamic complexity underlying the data. The experimentalist can use BIC along with plots of fitted parameters to adopt working models and to design further tests of these models.

The BIC rewards models that fit the data well and punishes extra free parameters to account for the ability of larger models to always fit data as well or better than smaller ones. The BIC relationship is given by equation 2:
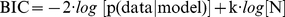
(2)where log p(data | model) is the log of the likelihood of the data under the maximum likelihood estimator (determined by maximizing the quantity in equation 1), k is the number of free parameters being fit, including transition rates, signal means, and standard deviations, and N is the length of the observed trace. The model with the lowest BIC provides the optimal fit of the models under comparison in terms of maximizing data likelihood and minimizing model complexity.

As a demonstration of how the BIC is used we examined the ability of the BIC to identify the correct three-state model out of six candidate models. Fig. 4A shows the three-state kinetic model used to generate mock traces. In this model, states 1 and 2 had emission properties identical to the states in Fig. 3A (also see Methods), and an equivalent of the SNR of 4 from that figure was used. State 3 was added with emission halfway between these states, resulting in an effective SNR between states of ∼2. Six candidate models were then fit to the traces. For models with three or more states, the interconnections between the states become an important consideration. In this example we consider three possible three-state models, but, for simplicity, we did not consider ones where only two distinct intensity levels are produced or where thermodynamically irreversible transitions occur.

Fig. 4C plots the determined BIC for each of the six models in order of increasing number of fitted parameters. Of all fits, the true model (3-linear) produces the lowest BIC. The 1- and 2-state fits have BICs that are much higher than for the other models, with all other models showing the expected trend of increasing BIC with an increasing number of free parameters. A simple way to visualize this analysis is to look at the difference between BIC values in a trace. Fig. 4D shows the difference in BIC value between all the fits and the true model. Although the absolute values of the BIC's vary from trace to trace (Fig. 4C), the 3-linear model is the minimum in all cases and thus the best fit to the data.

The ability of the BIC to correctly identify the true number of states depends strongly on the details of a system and experimental data. For instance, in the two-state system shown in Fig. 3C, a one-state model gives the best fit to the data below an SNR of 0.7 (opaque gray region), indicating no meaningful kinetic information can be extracted with such a small SNR. In Fig. S4 of Appendix S1 we examine the effect of state lifetime, trace length, and distinctness of intensity level for each state on the ability of the BIC to identify the true model. These examples highlight limitations that exist when trying to identify the best model and the need to use BIC in conjunction with and as a guide for additional experimental tests. The BIC can also be used to evaluate which noise model optimally fits the data (i.e., Poissonian or Gaussian), although we have not systematically investigated this behavior.

### Clustering Algorithms to Assess Heterogeneity in Single Molecule Data

The determination of kinetic and thermodynamic parameters from individual molecules provides sensitivity unmatched by bulk techniques. For many systems this sensitivity has revealed persistent long-lived differences in seemingly identical molecules –i.e., molecular heterogeneity [13], [15], [17], [64]. This type of direct observation is unique to single molecule measurements and has garnered much attention. There is evidence that the observed heterogeneity can arise from large barriers in deeply furrowed energy landscapes and from covalent differences between molecules in the population [13], [17], [65].

No standardized approach exists for assessing if the data from an experiment is stereotyped by heterogeneity. As a first-pass approach, SMART allows data to be plotted with statistically rigorous confidence intervals that can then be visually assessed for the degree to which the confidence intervals overlap or fall into well separated groups. SMART also implements a more systematic analysis tool that groups molecules into clusters based on the similarity of their inferred parameters and the uncertainty in inferring those parameters. This tool allows single molecule data for populations of molecules to be systematically and rapidly analyzed.

SMART allows the user to assess molecule-to-molecule variation by clustering up to three jointly inferred model parameters. The clustering algorithm in SMART takes as inputs the MLE model parameters and confidence bounds determined by an HMM fit for each trace in the data set. These parameters are then grouped by fitting models with different numbers of clusters. An expectation maximization algorithm is used to find the MLE fit for the cluster positions [50]. This task is accomplished by evaluating the likelihood of each trace arising from each cluster and then adjusting the cluster positions to maximize the expected likelihood of the data set. This computation can be completed quickly -in minutes on a desktop computer- by approximating the likelihood for each trace with a normal distribution. A detailed description of this algorithm and an analysis of this approximation is provided in Appendix S1. Key outputs that can be used to describe and evaluate the cluster fit include the cluster center positions, the probability that a trace resides within a cluster for each trace, the total size of the cluster (i.e., the number of traces expected to reside within the cluster), and the log likelihood or BIC for the data set under the cluster model. To demonstrate the utility of the SMART clustering algorithms for assessing heterogeneity in single molecule data, we first present simulated data for a hypothetical heterogeneous system. We then present actual smFRET data that was previously analyzed using a more time-consuming and less direct simulation approach.

To test the ability of the clustering approach to differentiate two populations of traces, traces were simulated (100 traces for each population) at four different SNRs ranging from 2 to 15 using the same relationship between SNR and trace length as in Fig. 3. For a given SNR, traces in the two populations are distinguishable because one of the rate constants differs by two-fold, which provides a rather stringent test of this algorithm (Fig. 5). Traces were then fit to two-state HMMs and traces at each SNR were fit with one to four clusters. Fig. 5B shows the determined cluster size for the two and three cluster fits. The one and four cluster fits are shown in Fig. S5A of Appendix S1; the one-cluster fits by definition contain all of the molecules analyzed. The fourth cluster did little to improve the overall fit as the size of the smallest two clusters in the four cluster fits was almost identical to the smallest cluster in the three cluster fits.

For the two-cluster fits, a SNR of around five or lower is needed for the two clusters to be nearly equally populated and thus to reflect the actual behavior of the two populations. For the simulations with SNRs of five or two, the determined clusters correctly partition ∼90% of the molecules and accurately determine the population centers (Fig. 5C, blued dots). This increased accuracy at lower SNR is a result of the relationship between SNR and the typical size of confidence bounds in inferred kinetic parameters shown in Fig. 3C above. Comparison of the two-cluster fit to the three-cluster fit shows only a minor change. The third cluster (Fig. 5B) is zero for SNRs five or higher and for an SNR of two it only accounts for 1 out of the 200 traces. Some molecules can populate the third cluster as a result of the stochastic variation in the inferred model parameters of the simulated traces. The marginal improvement seen for the addition of the third cluster and fourth cluster can be further evaluated using plots of the log of p(data | model) for the one to four cluster fits shown in Fig. S5B of Appendix S1. The value of the log likelihood for the data set increases as more clusters are added, and the leveling off of this increase is an indication that the correct number of clusters has been surpassed. This criterion further supports the cluster assignment suggested above by our analysis of the cluster size.

These results indicate the clustering approach can be a powerful tool for analyzing populations of molecules. The clustering approach directly relates uncertainties in parameters for individual molecules to the behavior of populations of molecules. Moreover, clustering eliminates many of the assumptions that would otherwise be used in the analysis of heterogeneity with simulations and can be completed rapidly.

To further test the utility of clustering we used SMART clustering to reanalyze part of the smFRET data from a prior RNA folding experiment. Fig. 6A shows a scatter plot of the folding and unfolding rate constants determined by fitting a two-state HMM to traces for folding of the P4–P6 domain of the *Tetrahymena* Group I intron in 2.5 mM Mg^2+^ [65]. Overlaid in red are simulations used to assess the heterogeneity in P4–P6. These simulations assume two populations of molecules with folding and unfolding rate constants differing slightly (*k*_f_ = 0.95/*k*_u_ = 0.80 and *k*_f_ = 1.35/*k*_u_ = 0.57 all [sec^−1^]) and with uncertainties due to SNR and trace length estimated from variation in the measured populations. These simulated distributions are able to account for 80% of all the molecules. This analysis in combination with other results supported the conclusion P4–P6 has relatively simple folding behavior, with most rate constants for individual molecules falling within a two-fold range [65]. Analysis of this type is a standard approach for evaluating heterogeneity in single molecule data and can give an assessment of the extent of heterogeneity in a system. However, the approach is time-consuming to implement and there are numerous assumptions that go into iteratively identifying the parameters used as inputs for the simulation and for evaluating the degree of overlap between the measured and simulated distributions.

These data were fit with one to five clusters using SMART (Fig. 6B & C). For the three-cluster fit the two main clusters account for 819 and 278 molecules (93%), while the remaining cluster contained only 79 of the molecules. An examination of the four-cluster fit reveals a similar result, with the two main clusters accounting for 556 and 514 molecules (91%) and the two minor clusters containing only 106. In the five-cluster fits the three smallest clusters contain 196 molecules with the two major clusters having a similar distribution as in the four-cluster fit. These results reveal a similar picture to the interpretation of the simulation shown in Fig. 6A. For instance, in the four-cluster fit the center of the two main clusters are (*k*_f_ = 0.91/*k*_u_ = 0.90 and *k*_f_ = 1.19/*k*_u_ = 0.51 all [sec^−1^]), which are nearly identical to the centers of the simulated distributions and these two clusters account for over 90% of the data. Since we do not know the true underlying distribution of P4–P6, in contrast to the simulations examined in Fig. 5, determining the optimal number of clusters will necessarily require an evaluation of the many possible sources that can contribute to the distribution in the data. For P4–P6 being able to account for 90% of the data with two kinetically similar populations of molecules is suggestive of a simple folding landscape for P4–P6. This is particularly the case considering that a small amount of remaining covalent heterogeneity could be the source for the variation seen in the remaining population of molecules. However, in stark contrast to the prior simulation analysis (Fig. 6A), this result is arrived at quickly, naturally, and without the need for extensive user input.

## Discussion

SMART provides a solution for contemporary challenges in the analysis of single molecule data, providing an ease of use, rigorous statistics, a semi-automated means to distinguish models, a convenient format for storing and sharing data and is freely accessible.

Single molecule approaches have enormous power to delve deeply into molecular mechanisms, but single molecule data also have many sources of uncertainties and can be unwieldy to manipulate and analyze. SMART aids in experimental design, revealing a counter-intuitive increase in data quality with lower SNRs because this decrease typically allows longer observation of individual molecules. Fitting the individual data traces obtained to HMMs is necessary to deal with the inherent stochastic variations. The HMM fitting algorithms in SMART are a more general implementation than have been routinely applied to single molecule data, allowing fits with multiple data types and with states having non-unique emissions. SMART also allows a thermodynamic constraint to be imposed and confidence intervals to be calculated for inferred parameters. The confidence intervals obtained in SMART allow a common and vexing issue in single molecule work to be addressed: to what extent do the individual molecules exhibit identical versus distinct behavior? The degree of overlap between the confidence intervals of inferred parameters provides a readout of how similar or different individual molecules are, and the clustering algorithms in SMART provide a natural and intuitive means for identifying groups of similar molecules.

In addition to providing a comprehensive and statistically rigorous means of analyzing single molecule data, SMART represents a step toward standardization of single molecule data. All the analysis tools in SMART are accessible through a graphical interface, allowing everything from specifying the model to be fit to the data, to inspection of fitted parameters, and clustering groups of similar molecules. The integration of commonly used functions speeds data analysis and allows investigators to focus on the design and optimization of experiments instead of the implementation of analysis protocols. The adoption of a common data analysis format should facilitate sharing of raw data and the critical assessment and re-assessment of analyses from other investigators, analogous to advances from standardization of X-ray structural, microarray, and other genomic data.

## Methods

See Appendix S1 for detailed descriptions of HMM and clustering fitting procedures.

Numerical simulations for Figs. 1, 3, 4, and 5 required sampling from a hidden Markov model (HMM) with a specified transition matrix A and emissions distribution. We first obtained a sequence of hidden states by sampling from the stationary distribution of the Markov chain and then repeatedly choosing a next state according to the transition probabilities specified by A. We then generated a sequence of emissions conditionally independent of each other given the hidden state. The emissions distributions we sampled from were the Poisson distribution with a specified mean for Figs. 1, 3, 4, and 5. We sampled from a single Poisson-distributed channel in Figs. 3, 4, and 5. We sampled from two Poisson-distributed, conditionally independent of the hidden state channels in Fig. 1 to simulate the availability of donor and acceptor fluorescence data. We did not simulate FRET directly or attempt to fit FRET traces, because we assume that the raw donor and acceptor traces are available in an experiment and because the ratio of two Poisson-distributed signals follows a ratio distribution and is not normal or Poisson. As noted above, more rigorous and accurate analyses can be carried out using the actual emission data from each channel.

These simulations required a choice of trace length T and signal to noise ratio (SNR). When we varied these parameters, as in Figs. 3 and 5, we held their product constant, T*SNR = c, to simulate the effect of photobleaching. Thus, higher optical power results in higher SNR but faster dye photobleaching and lower dye lifetime T. A higher constant c corresponds to a higher total mean number of photons a dye emits before photobleaching. SNR increases in proportion to the square root of the number of channels, so two identical independent intensity channels with SNR 1 would produce overall SNR √2.

For a single Poisson-distributed channel with means μ_1_ and μ_2_ in states 1 and 2, as in Figs. 3, 4, and 5, SNR is defined by equation 3.
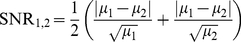
(3)For example a Poisson-distributed channel with mean intensities of 100 and 110 in states 1 and 2, respectively, corresponds to a SNR of about 1.

## Supporting Information

Appendix S1 **Includes supporting figures and supporting methods.** Supporting methods provides a detailed description of HMM specification, HMM fitting procedures, confidence interval determination, model selection, cluster determination, cluster selection and conversion from continuous to discrete time models.
(PDF)
