# Supplementary material for: Single Molecule Analysis Research Tool (SMART): An Integrated Approach for Analyzing Single Molecule Data
Source: PLoS One. 2012 Feb 20;7(2):e30024. doi: 10.1371/journal.pone.0030024 (PMC3282690; doi:10.1371/journal.pone.0030024)
Supplement: Appendix S1 — Includes supporting figures and supporting methods. Supporting methods provides a detailed description of HMM specification, HMM fitting procedures, confidence interval determination, model selection, cluster determination, cluster selection and conversion from continuous to discrete time models. (PDF) [file pone.0030024.s001.pdf]

Single Molecule Analysis Research Tool (SMART): An integrated approach for analyzing single molecule data

Max Greenfeld[1,2,#], Dmitri S. Pavlichin[3,#], Hideo Mabuchi[4,*] Daniel Herschlag[1,2,*]

1 Dept. of Chemical Engineering, Stanford University, Stanford, CA, USA
2 Dept. of Biochemistry, Stanford University, Stanford, CA, USA
3 Dept. of Physics, Stanford University, Stanford, CA, USA
4 Dept. of Applied Physics, Stanford University, Stanford, CA, USA

# These authors contributed equally

∗ E-mail: Corresponding herschla@stanford.edu
∗ E-mail: Corresponding hmabuchi@stanford.edu

Supporting Information

Supporting Figures

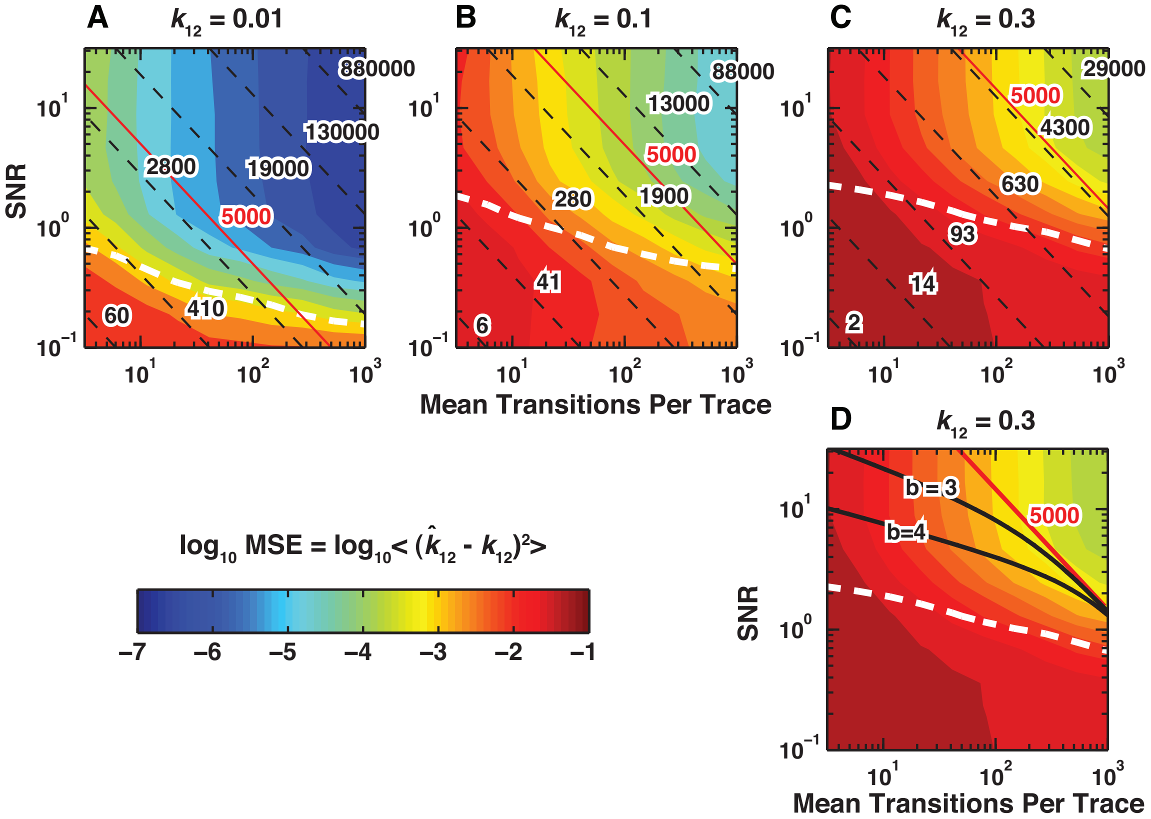

Figure S1: Performance of HMM fits depends jointly on SNR, trace length, and rate constants. The plots are contoured, nearest-neighbor smoothed plots of mean square error (MSE) for the estimator of k_{12}, $\log_{10}\mathrm{MSE} = \log_{10}\langle(\hat{k}_{12} - k_{12})^2\rangle$ sampled on a 10 by 10 grid of SNR and trace length values for three values of k_{12} (and $k_{21} = k_{12}$). Both longer observation times (higher mean number of transitions per trace) and higher SNR reduce the mean square error in the estimator of k_{12} produced by the fit. Dashed black lines indicate regions where the product of the SNR and the trace length is constant, with the value of this constant shown in black next to the lines. The solid red line indicates the region of SNR$\times$T = 5000, the value used in the body of the paper. For an experiment constrained to one of the dashed lines, the minimum MSE is obtained when the dashed line is tangent to the lowest-value constant MSE line. For SNR$\times$T = 5000, the MSE-optimal SNR is (A) $\sim$1 for $k_{12} = 0.01$, (B) $\sim$2 for $k_{12} = 0.1$, (C) $\sim$4 for $k_{12} = 0.3$. The white dashed line indicates the region below which a one-state fit achieves a lower BIC for more than half the simulated traces. (D) The effect of two alternative photobleaching models on the accuracy of inferred rates are plotted for the condition examined in Fig. 3C. The photobleaching models are described by the relationship T(SNR+0.001 SNR^b)=5000, where b = 3 or 4. This relationship was chosen to reflect the faster than expected photobleaching at high SNR that can occur in some experiments.

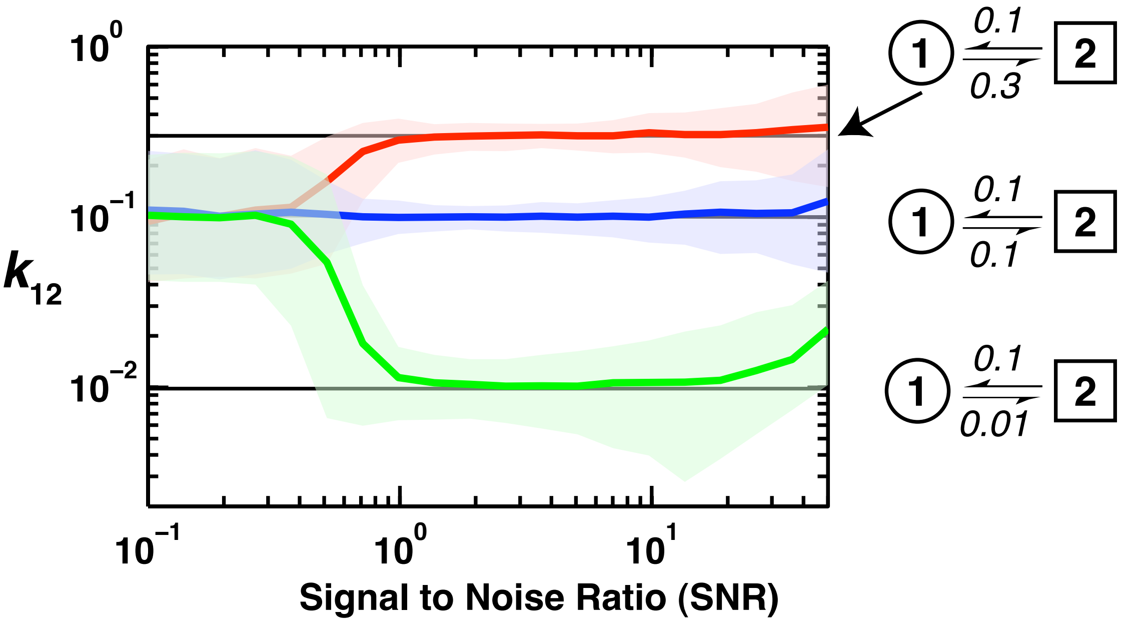

Figure S2: HMMs are not an unbiased estimator of rate constants. Traces were simulated for the three kinetic models and SNRs were varied according to the emission model described in the Methods. The colored swaths represent the region that bound 90% of the determined rate constants from the 1000 simulated traces that were analyzed by fitting to two state HMMs. This result clearly shows at an SNR of ∼1 that maximum likelihood estimation for HMMs does not produce an unbiased estimator for the model parameters as all models converge to the value of $k_{12} = 0.1$.

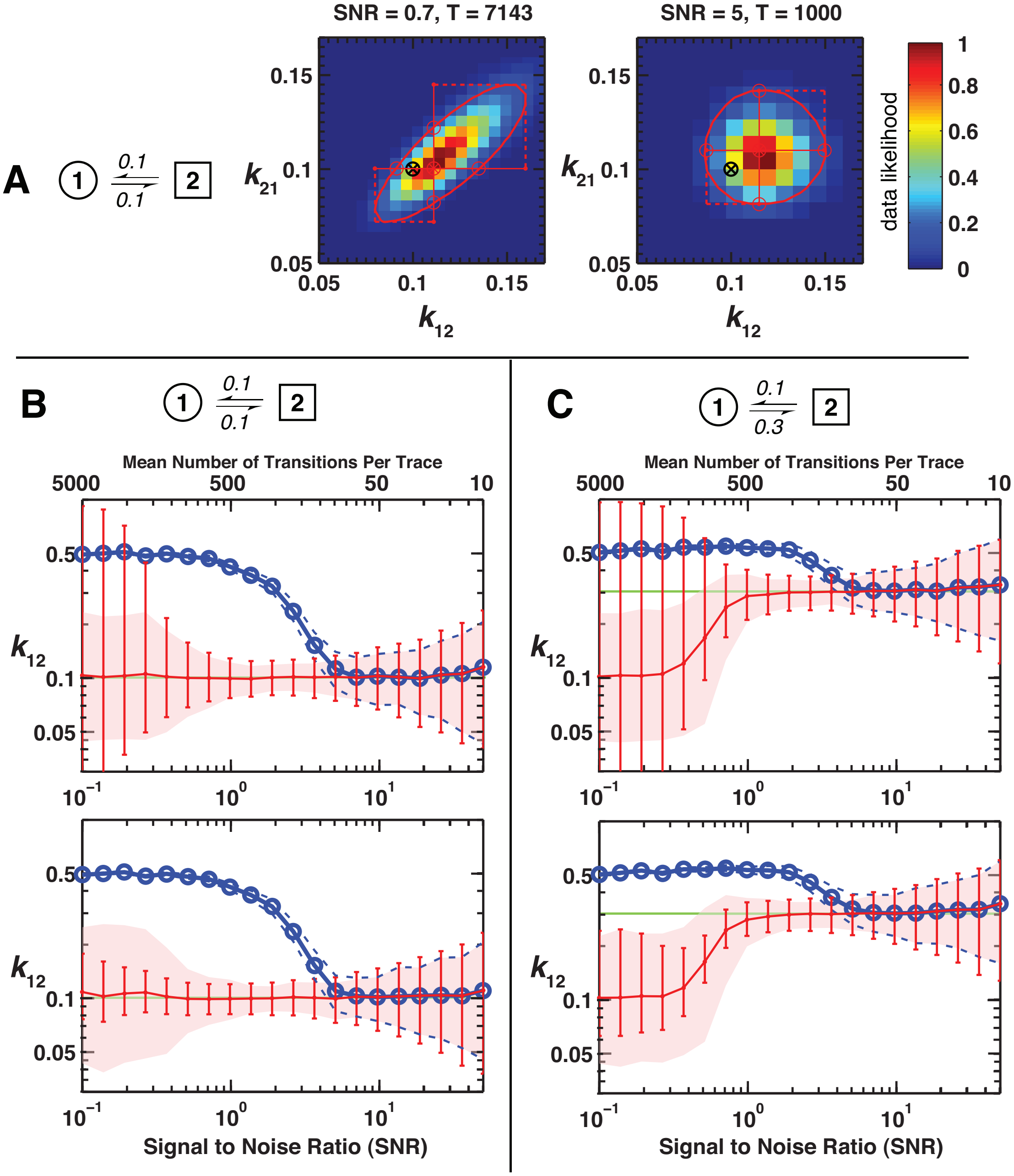

A
0.1
0.1
1
2
SNR = 0.7, T = 7143
SNR = 5, T = 1000
k_{21}
k_{12}
0.05
0.1
0.15
data likelihood
0
0.2
0.4
0.6
0.8
1
B
C
0.3
Mean Number of Transitions Per Trace
5000
500
50
10
k_{12}
0.5
10^{-1}
10^{0}
10^{1}
Signal to Noise Ratio (SNR)

Figure S3: HMM algorithms in SMART can determine one-parameter and two-parameter confidence intervals. In some regimes examination of the two-parameter fits give a better approximation of the true uncertainty in the determined rate constants. (A) Depiction of pairwise confidence intervals calculated for $k_{12} = k_{21} = 0.1$ and SNRs of 0.7 and 5 for two particular traces and determined according to the emissions model described in the Methods. The black circle/cross indicates the true transition probabilities. Heat map colors indicate the likelihood of the trace given a particular value of k_{12} and k_{21}, normalized to 1 at the maximum likelihood estimator. The red oval indicates the 90% pairwise confidence interval. The red dashed lines are left-, right-, bottom-, and top-most tangents to the oval. The red circles indicate the one-dimensional projection confidence bounds obtained by varying only one parameter at a time. At an SNR of 0.7 the uncertainties are correlated more than at an SNR of 5. This causes underestimation of uncertainties in the one-dimensional projections of confidence intervals at SNR values below approximately 1 (Fig. 3). (B) To compare the uncertainties depicted in (A) over a range of SNR values, the left- and right-most points on the oval were compared to the simple one dimensional projections. The red swath and region bounded by the blue dashed line represent the regions that bound 90% of the determined rate constants from the 500 simulated traces that were analyzed by fitting to two-state HMMs or with thresholding, respectively. The red error bars on the top plot show the confidence intervals determined by the left- and right-most points on the oval (Fig. S3 A red dashed lines). The bottom plot shows the one-dimensional projection of uncertainties (Fig. S3 A red circles). In the low SNR regime the left- and right-most points on the oval are a better approximation of the measurement uncertainties compared to the one-dimensional projections. Overestimation of uncertainties (red error bars compared to the red swath) can occur when the normal approximation to the likelihood function begins to break down. (C) Same as in (B) but where the kinetic model is $k_{12} = 0.3$ and $k_{21} = 0.1$.

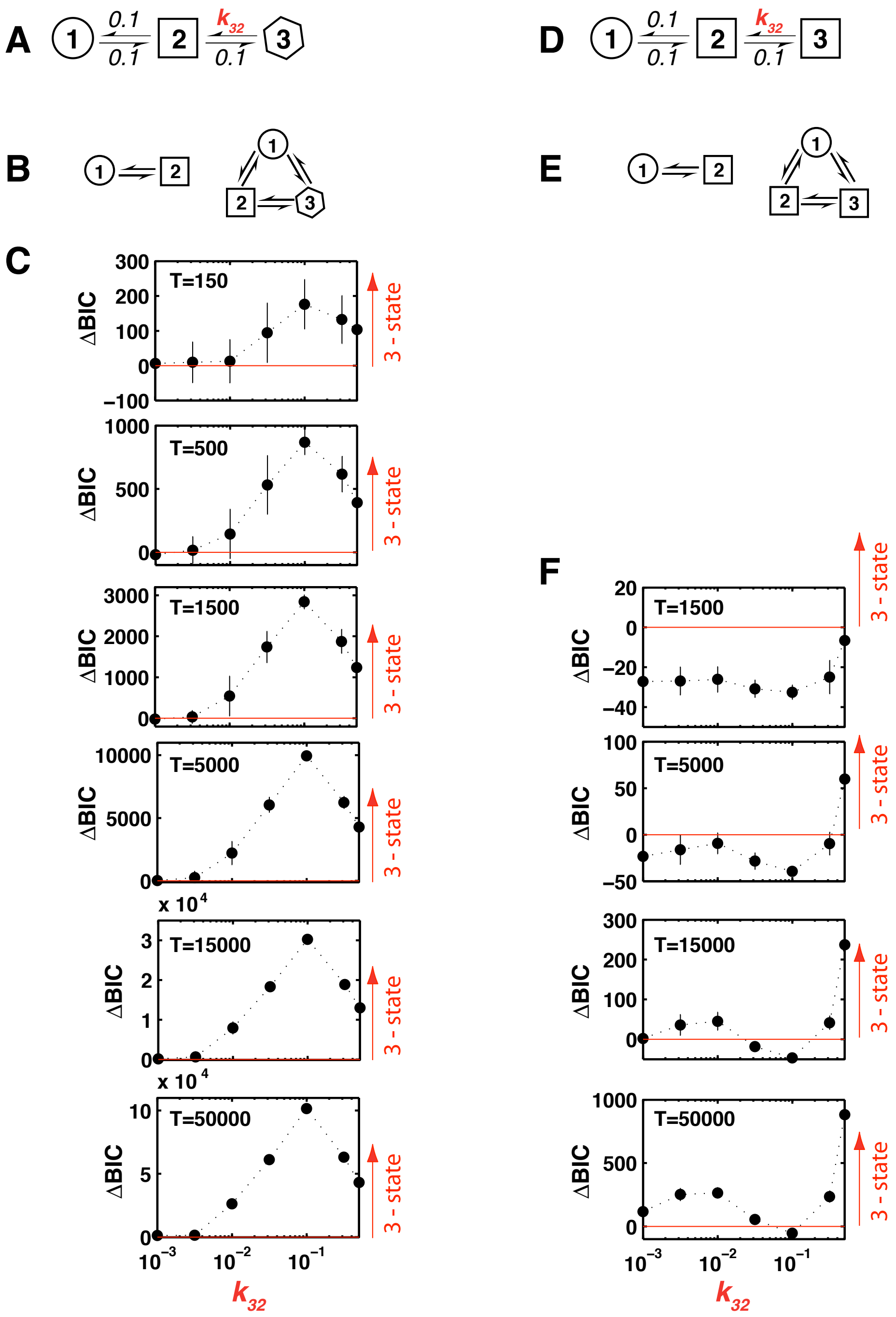
A
D
B
E
C
F
T=150
T=500
T=1500
T=5000
T=15000
T=50000
ΔBIC
3 - state
x 10^4
k_32
0.1

Figure S4: Additional simulations mapping out the ability of the BIC to identify the presence of a third state. In these simulations we considered the effect of transition rate, trace length and the distinguishability of states on the ability of the BIC to identify the presence of a third state in a trace. We only considered the difference in the BICs between the two- and three-state models shown in B and E. Positive values indicate the third state is identified and the larger the value the more distinguishable the state is. As demonstrated in Fig. 5 the difference among the various three-state models and the four-state model is quite small relative to the one- and two-state models. Therefore models that are strongly three-state by this test should show the same trend for the other three-state and four-state models shown in Fig. 5. (A) Three-state traces were generated from a model that in the limit of large k_{32} become equivalent to the two-state traces with an SNR of 4 and $k_{12} = k_{21} = 0$. When the time spent in the third state is significant, its emission intensity is midway between that of the low and high intensity emission. (B) Simulated traces were fit to the most general two- and three-state models where all transitions were allowed and each state could have a unique emissions distribution. (C) For trace lengths varying from 150 to 50,000 the mean difference in the $\Delta\text{BIC} = (\text{BIC}_3 - \text{BIC}_2)$ value and standard deviation determined from fits to 50 simulated traces are plotted for values of k_{32} varying from 0.001 to 0.5. The point where all the rate constants are equal is the point where the models were most distinguishable. In almost all instances the third state was detected. (D) Simulation conditions were identical to those used in A, except the third state had an emissions distribution identical to the second state, as shown by the square surrounding states 2 and 3 compared to only state 2 in part A. When all rate constants are equal this model is indistinguishable from a two-state model. (E) Same as in (B) except the third state does not have an unique emissions intensity, as shown by the square surrounding the states 2 and 3 compared to only state 2 in part A. (F) For trace lengths varying from 1500 to 50,000 the mean difference in the $\text{BIC}_1 - \text{BIC}_2$ value and standard deviation determined from fits to 50 simulated traces are plotted for values of k_{32} varying from 0.001 to 0.5. For about half of the conditions examined the third state was not distinguishable, as indicated by the negative difference in the BICs. Moreover the differences are relatively small compared to the case when the states have distinguishable means. These results indicate a third state that does not have a unique emission intensity can be identified using this technique, but the sensitivity is significantly reduced from situations where the state does produce a unique intensity. An additional consideration for fits to models of this type is that they are under-constrained and no unique set of rate constants provides the best fit. Rather, a family of rate constants fit the traces equally well; further discussion of this point is in Supporting Methods.

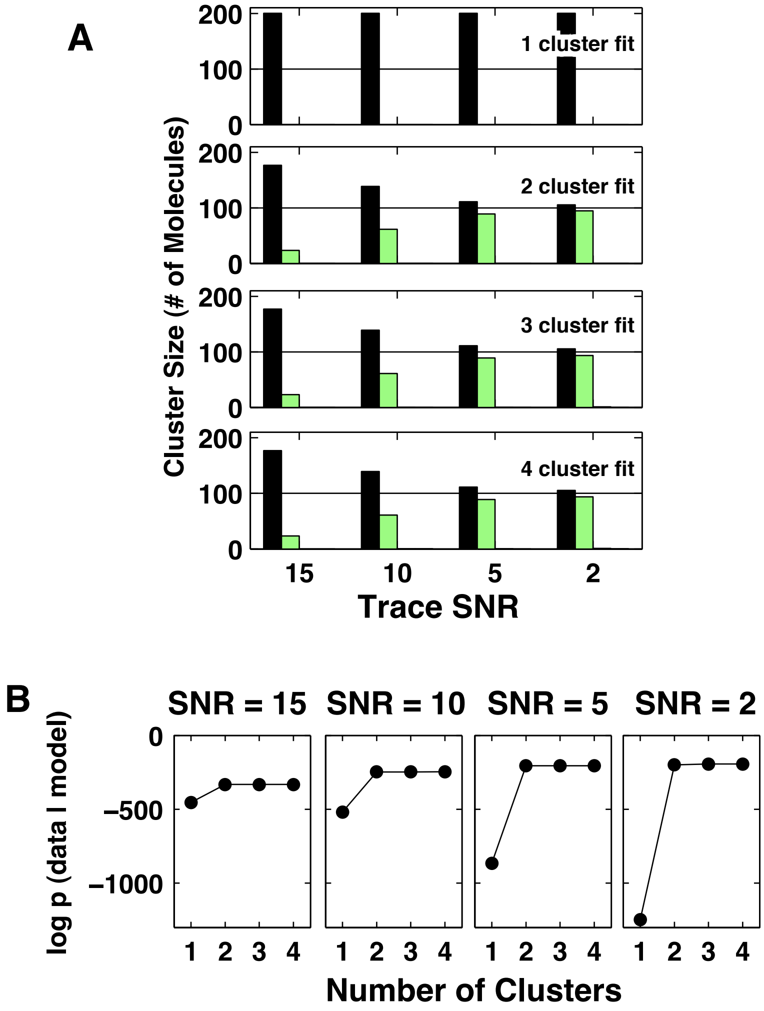
A
1 cluster fit
2 cluster fit
3 cluster fit
4 cluster fit
Cluster Size (# of Molecules)
Trace SNR
B
SNR = 15
SNR = 10
SNR = 5
SNR = 2
log p (data I model)
Number of Clusters

Figure S5: Analysis of cluster size and log likelihood ($\log p(\text{data} \mid \text{model})$) cluster selection criteria for clustering two non-exchanging populations of molecules. Traces with SNRs of 2, 5, 10 and 15 were generated from two non-exchanging pools of molecules (100 traces each) with one transition rate differing by two fold. The traces were fit to two-state HMM models and subjected to clustering analysis in SMART, traces were fit with 1 to 4 clusters. (A) The cluster size (each bar corresponds to a cluster) for each cluster is shown. The black and green bars correspond to an individual cluster size at the indicated SNR, the bars corresponding to the third and fourth cluster in the 3- and 4- cluster fits are not visible due to their small size. (B) In addition to cluster size (see main text) the log likelihood is a useful model selection criteria. The log likelihood always increases as more states are added to a fit. When little change is seen in the log likelihood with added states, this indicates that the states are not necessary to fit the data well. A large change between the one- and two-state fits is seen at all SNR, with much smaller changes seen for the higher-order fits. While the log likelihood should always increase with an increase in the number of fitted parameters, some uncertainty (due to variation in initial conditions and propensity of the algorithm to converge to a local minimum) is associated with the final fitted values. As discussed in the main text this uncertainty is difficult to quantify.

Supporting Methods

Hidden Markov models for multichannel single molecule signals

This section exists mostly to establish notation. A discrete-time, K-state HMM describes the stochastic generation of an unobserved sequence of hidden states $(x_1, \ldots, x_N)$ and a corresponding observed sequence of emissions $(y_1, \ldots, y_N)$. A complete description of a HMM requires a $K \times K$ transition matrix A

$$(A)_{ij} = p_{i \to j} = \mathrm{P}(x_{t+1} = j | x_t = i) \tag{1}$$

and any parameters necessary to calculate the emission probabilities $\mathrm{P}(y_t | x_t = k)$. the case of normally distributed emission values, for example, we must specify a set of means $\mu_1, \ldots, \mu_K$ and variances $\sigma_1^2, \ldots, \sigma_K^2$. When the observed emissions are in the continuous space of reals, as in the normal case, $\mathrm{P}(y|x)$ is a sample from a probability density function (pdf) and is not a probability, but we shall keep the $\mathrm{P}(y|x)$ notation for clarity. It is useful to consider a diagonal $K \times K$ emission matrix E_t, whose elements for each time t are

$$(E_t)_{ii} = \mathrm{P}(y_t | x_t = i) \tag{2}$$

If the observed data $y_t = (y_{t,1}, \ldots, y_{t,C})$ comes from C channels, such as a donor and acceptor channel in the FRET case, we can obtain an overall emission matrix by multiplying the emission matrices of each channel

$$E_t = \prod_{c=1}^{C} E_{t,c} \tag{3}$$

This expression is valid only if the multiple channels emit independently for every hidden state of the model. Otherwise, we can obtain an emission matrix by sampling from the joint pdf of the multiple channels, say, a multivariate normal pdf with a covariance matrix for the different channels.

Data likelihood computation

Suppose that at time $t = 0$ our best guess for the state of the system is the vector $f_0[i] = \mathrm{P}(x_0 = i)$. If we then watch the system for N steps and record N observations

(the data), we can compute the probability of seeing these observations given our knowledge of the HMM parameters (the model):

$$p(\text{data} \mid \text{model}) = \vec{1}_K \cdot \left(\prod_{t=1}^{N} E_t A^{\mathrm{T}} \right) \vec{f}_0 \tag{4}$$

We must take the dot product with $\vec{1}_K$, the vector of K 1's, in order to sum over the K possible final states of the system.

Computing the maximum likelihood estimator for model parameters

The maximum likelihood estimator (MLE) for the transition rates and emission parameters of a HMM maximizes the quantity in equation 4 as a function of the model parameters. We calculate the MLE for a K-state HMM for a single data trace by the Baum-Welch algorithm (BW). BW is an expectation-maximization algorithm that iteratively computes expected log likelihood of the data given the model (expectation) and updates the model parameters to maximize this expected likelihood (maximization). We run the algorithm until numerical convergence of the model parameters.

The MLE for a HMM is not unique, in that we could re-label all of the states for an MLE to obtain another MLE with permuted transition and emissions matrices. This possibility prevents us from directly comparing inferred model parameters for two different traces, since two apparently different fits may actually be similar after state re-labeling. To break this symmetry we enforce an arbitrary constraint during the optimization process that the states are numbered in order of increasing signal mean.

If our model contains multiple states with identical emissions distributions, the transition rates may not be identifiable. That is, a continuum of rates a_{ij} may provide an equally good fit to the data. In this case, our fitting algorithm will output one set of parameters that fit the data well but provide no information about the set of all parameters that fit equally well. It may still be useful to fit these underconstrained models in inferring the number of hidden states (see "Inferring the number of hidden states" section below). For systems that are in thermodynamic equilibrium (satisfy detailed balance) [1] shows that we can fit a unique set of rates if we assume no interconversion between any states that have identical emissions distributions, and that this assumption does not hurt the quality of the fit (does not reduce $p(\text{data} \mid \text{model})$).

Baum-Welch algorithm details

Given a sequence of observations $(y_1, \ldots, y_N)$ and an initial guess for the HMM transition rates and emission parameters, which give us an initial transition matrix $\hat{A}$ and emission matrices $\hat{E}_{t=1,\ldots,N}$, BW recursively computes three K by 1 vector quantities for each time step. $\vec{f}, \vec{b}, \vec{p}$ are proportional to the forward, backward, and posterior probabilities:

$$\begin{array}{lllll} \vec{f_t} & \sim & \mathrm{P}\left(x_t = k, y_1, \ldots, y_t\right)_{k=1\ldots K} & \sim & \left(\hat{E}_t\, \hat{A}^{\mathrm{T}}\right) \vec{f}_{t-1} \\ \vec{b_t} & \sim & \mathrm{P}\left(y_{t+1}, \ldots, y_N | x_t = k\right)_{k=1\ldots K} & \sim & \left(\hat{A}\, \hat{E}_{t+1}\right) \vec{b}_{t+1} \\ \vec{p_t} & \sim & \mathrm{P}\left(x_t = k | y_1, \ldots, y_N\right)_{k=1\ldots K} & \sim & \vec{f_t} \odot \vec{b_t} \end{array} \quad (5)$$

To avoid numerical underflow we normalize $\vec{f_t}$ and $\vec{b_t}$ at each time t, hence the $\sim$ notation. $\odot$ denotes the pointwise product of two vectors: $\vec{a} \odot \vec{b}[i] = \vec{a}[i] \cdot \vec{b}[i]$. These computations require initial conditions; a reasonable choice for $\vec{f_0}$ is the steady-state occupation distribution implied by $\hat{A}$, while $\vec{b}_N = (1, \ldots, 1)^{\mathrm{T}}$ by definition. The most probable state at each time t the data and our guess for the model is then

$$\hat{x}_t = \arg \max_{k=1\ldots K} \vec{p_t}[k] \quad (6)$$

Once computed, the quantities in equation 5 can be used to update the current guess for the transition matrix $\hat{A}$ and the emission parameters used to compute $\hat{E}_{t=1,\ldots,N}$. For example, for a single poissonian channel, the MLE mean signal value in state i $_i$ is given by

$$\hat{\lambda}_i = \frac{\sum_{t=1}^{N} y_t\, \vec{p_t}[i]}{\sum_{t=1}^{N} \vec{p_t}[i]} \quad (7)$$

Inferring the number of hidden states

The log-likelihood of the observations given the maximum likelihood K-state HMM is a nondecreasing function of K. A common method to select the number of hidden states is to penalize additional degrees of freedom by choosing a model that minimizes the Bayesian Information Criterion (BIC), defined by

$$\mathrm{BIC} = -2 \log p(\text{data} \mid \text{model}) + d \log N \quad (8)$$

where d is the number of degrees of freedom for the HMM and N is the number of observations. For example, a two-state HMM with a single poissonian emissions channel has four free parameters (two rates and two signal means). Our implementation calculates and plots the log likelihood and BIC for HMMs with up to some

user-defined maximum number of states.

Fitting a model with parameters that are not identifiable when multiple states have identical emissions distributions returns one of a continuum of models that all fit the observations equally well. Fitting an underconstrained model may still be useful, in that $p(\text{data} \mid \text{model})$ will still increase as we fit models with increasingly many hidden states. For example, we may observe a dwell time distribution in a low FRET state that is well fit by a mixture of two exponential distributions, so we may infer that there are two hidden states with an identical emissions distribution, even if we are unable to uniquely determine the interconversion rates between these two states.

Calculation of confidence region for model parameters

After obtaining an MLE estimator for the HMM parameters, we vary the model parameters in a region near the MLE and record decreases in the log likelihood (equation 4). We can vary one parameter while holding the others fixed, or vary a subset of them together. We obtain likelihood ratio confidence bounds by choosing a threshold for the minimum value of the data likelihood that all models inside the confidence bound satisfy.

We follow the approach of Giudici et al. [2]. Let θ in $\mathbb{R}^d$ be the vector of true, unknown model parameters, and $_{\text{MLE}}$ in $\mathbb{R}^d$ the d-dimensional MLE for the model parameters found by BW. We have null hypotheses of the form $\theta = \theta'$ with likelihood ratio (LR) test statistic

$$\text{LR} = 2\left(\log p(\text{data } |\theta_{\text{MLE}}) - \log p(\text{data } |\theta')\right) \tag{9}$$

For large observation number N and under the null hypothesis LR is approximately χ^2_d-distributed. We reject all models for which $\text{LR} > \chi^2_d(1-\alpha)$ to form our confidence bound, where a typical choice for α is 0.05 or 0.01. For example, in inferring the two transition rates $a_{1,2}$ and $a_{2,1}$ for a two-state HMM with known emissions distribution, we would reject all models such that

$$2\left(\log p(\text{data } |\theta_{\text{MLE}}) - \log p(\text{data } |\theta')\right) > \chi^2_2(1-\alpha) \Rightarrow \frac{p(\text{data } |a'_{1,2}, a'_{2,1})}{p(\text{data } |a^{\text{MLE}}_{1,2}, a^{\text{MLE}}_{2,1})} < \alpha \tag{10}$$

Allowing multiple parameters to vary together is computationally expensive, as the time and space in memory needed to record a d-dimensional likelihood region scale

as $O(md)$, where m is the meshsize - the number of likelihood samples per parameter in the region of the MLE. We thus vary only up to two parameters together, and potentially overlook higher-dimensional structure in the log likelihood near the MLE.

Clustering

Log likelihood for multiple traces under one model

If we are working with a set of multiple traces, as we would when combining the data of multiple molecules, we can compute a likelihood for all of them if we assume that the traces are generated independently. For N_T independent traces, the log likelihood for a particular model with parameter vector ϕ is

$$\log p(\text{all traces } |\phi) = \sum_{i=1}^{N_\mathrm{T}} \log p(\text{trace}_i \ |\phi) \tag{11}$$

Log likelihood for multiple traces under a mixture of different models

For clustering analysis we assume that the traces are independently generated from a mixture of C models indexed by parameter vectors $\Phi = (\phi_1, \ldots, \phi_C)$ and that we have found for each of the N_T traces a maximum likelihood estimator of the model parameters $(\theta_1^{\mathrm{MLE}}, \ldots, \theta_{N_\mathrm{T}}^{\mathrm{MLE}})$ obtained by Baum-Welch. The t-th trace has an unknown label λ_t in $\{1, \ldots, C\}$ corresponding to the mixture component to which it belongs. Each model i in the mixture generates a fraction η_i of the traces. In this case the log likelihood for the mixture model is

$$\log p(\text{all traces } |\Phi, \eta) = \sum_{i=1}^{N_\mathrm{T}} \log \left(\sum_{j=1}^{C} p(\text{trace}_i, \lambda_i = j | \Phi, \eta) \right) \tag{12}$$

We are excluding the trace length, which varies trace to trace and is determined by experimental conditions like optical power, from our clustering analysis. This is because we do not have a general model for the trace length distribution as a function of experimental parameters. In the text, we held the product of SNR and trace length constant, but this is a phenomenological model that we used to illustrate the tradeoff between higher SNR and longer observation time, and this product relationship would need to be verified for data. Our implementation of clustering would thus miss groups of traces that differ only in their length distribution.

Now we attempt to maximize the likelihood by maximizing the expected likelihood, by iteratively maximizing the quantity

$$Q((\Phi'\eta'),(\Phi,\eta)) = \sum_{i=1}^{N_\mathrm{T}} \sum_{j=1}^{C} p(\lambda_i = j|\mathrm{trace}_i, \Phi, \eta) \log \frac{p(\mathrm{trace}_i, \lambda_i = j, \Phi', \eta')}{p(\lambda_i = j|\mathrm{trace}_i, \Phi, \eta)} \tag{13}$$

as a function of $(\Phi'\eta')$. We will approximate $p(\mathrm{trace}_i, \lambda_i = j|\Phi, \eta)$ by the probability density function for a multivariate normal distribution, so that we can perform the optimization of Q as a function of $(\Phi'\eta')$ by setting

$$\phi'_j = \frac{\sum_{i=1}^{N_\mathrm{T}} p(\lambda_i = j|\mathrm{trace}_i, \Phi, \eta)\ \theta_i^{\mathrm{MLE}}}{\sum_{i=1}^{N_\mathrm{T}} p(\lambda_i = j|\mathrm{trace}_i, \Phi, \eta)} \tag{14}$$

$$\eta'_j \sim \sum_{i=1}^{N_\mathrm{T}} p(\lambda_i = j|\mathrm{trace}_i, \phi_j, \eta_j) \tag{15}$$

The vector η' is normalized to sum to 1. To compute the above quantities we use Bayes rule to write

$$p(\lambda_i = j|\mathrm{trace}_i, \Phi, \eta) \sim p(\mathrm{trace}_i|\phi_j)\ \eta_j \tag{16}$$

The vector $p(\lambda|\mathrm{trace}_i, \Phi, \eta)$ is normalized to sum to 1. We repeat this update rule until numerical convergence of (Φ', η').

Quick computation of data likelihood for cluster model

To evaluate the quantities in equations 14,15 we must repeatedly compute the quantity $p(\mathrm{trace}_i|\phi_j)$ for each of N_T traces for each of C clusters for each iteration until numerical convergence of (Φ', η'). Using the expression in equation 4 to do this is slow, as each evaluation of equation 4 requires us to look at the data in the trace. Instead we approximate this likelihood with a normal distribution. This assumption is justified for long traces in [3].

$$p(\mathrm{trace}_i|\phi) \sim \mathrm{N}(\theta_i^{\mathrm{MLE}}, K) \tag{17}$$

To find the covariance matrix K we evaluate $p(\mathrm{trace}_i|\phi_j)$ the slow way (using equation 4) at several points near θ_i^{MLE} and then run a numerical solver in MATLAB to fit these values to a multivariate normal distribution. Once we have computed K, we can evaluate $p(\mathrm{trace}_i|\phi_j)$ at additional points ϕ without looking at the trace data again by evaluating the pdf of this distribution at ϕ. This method reduces to k-means clustering if we reduce the entries of K by a large factor, so that one of $p(\mathrm{trace}_i|\phi_j)$ is much larger than the others for some cluster j.

Cluster size selection

To aid the user in estimating the number of clusters, we compute both a Bayesian information criterion (BIC) and the cluster size distribution as a function of total cluster number. To compute the BIC, we compute the likelihood for the dataset under the mixture model (Φ, η) using equation 12 and then compute the BIC:

$$\text{BIC}(\Phi, \eta) = -2 \log p(\text{all traces } |\Phi, \vec{\eta}) + C \cdot d \cdot \log N_{\text{T}} \tag{18}$$

Where C is the number of clusters, N_{T} is the number of traces, and d is the number of parameters along which we are clustering (our implementation restricts d to at most 3).

A visually useful way to select the number of clusters is to keep increasing the total cluster number until new clusters have hardly any members. To compute the cluster size distribution, we assign each trace to its nearest cluster by choosing j to maximize the quantity in equation 16 for each trace and count up the number of traces in each cluster.

$$\text{size of cluster } j = |\{\text{trace}_i | j = \arg\max_k p(\lambda_i = k|\text{trace}_i, \Phi, \eta)\}| \tag{19}$$

Thermodynamic constraint

In general the fit HMM of the Baum-Welch algorithm need not satisfy detailed balance. One may have good reason to believe the system in question is in thermal equilibrium and wish to impose this constraint. Even if the data arises from a model in thermal equilibrium, the optimization algorithm is likely to output rates that almost but not exactly satisfy detailed balance, while the quality of fit would not suffer if we were to insist on this condition being exactly (within numerical tolerance) satisfied during optimization. We describe our imposition of this constraint in the optimization routine.

A system in thermal equilibrium satisfies detailed balance, which may be stated in two equivalent ways:

$$\pi_i \; a_{i,j} = \pi_j \; a_{j,i} \forall \text{ states } i, j \tag{20}$$

$$a_{c_1,c_2} a_{c_2,c_3} \cdots a_{c_k,c_1} = a_{c_1,c_k} \cdots a_{c_3,c_2} a_{c_2,c_1} \forall \text{ cycles } (c_1, \dots, c_k) \tag{21}$$

Where π is the stationary distribution of the Markov chain with transition matrix $(A)_{i,j} = a_{i,j}$ and $(c_1, \dots, c_k)$ is a cycle of states of the Markov chain.

We implement an approach in the spirit of equation 20. The idea is to impose

detailed balance by averaging the transition matrix with its time-reversed version A_{rev}, much as we can symmetrize a matrix by averaging it with its transpose. We obtain A_{rev} by

$$A_{\text{rev}\ j,i} = P(x_t = i|x_{t+1} = j) = \frac{P(x_{t+1} = j|x_t = i)\ P(x_t = i)}{P(x_{t+1} = j)} = a_{i,j}\frac{\pi_i}{\pi_j} \tag{22}$$

And obtain a transition matrix that satisfies detailed balance A_{db} by

$$A_{\text{db}} = \frac{A + A_{\text{rev}}}{2} \tag{23}$$

We can verify that A_{db} satisfies detailed balance and has the same stationary distribution π as A. A_{db} has the nice properties that $a_{i,j} = a_{j,i} = 0 \Rightarrow a_{\text{db},\ i,j} = a_{\text{db},\ j,i} = 0$, that the state lifetimes implied by A and A_{db} are the same and that A_{db} is not far from A if A is not far from satisfying detailed balance. In this way we do not need to fix an arbitrary choice of rates as a function of the others or enumerate the cycles of our chain in the correct order while taking into account rates that are 0, and are not susceptible to the large numerical deviations from A that may occur when one rate is small.

This method does not hold a subset of the rates fixed to satisfy detailed balance. This complicates interpretation of confidence bounds for the rates, since we can not vary just one rate in the transition matrix; all rates are altered to satisfy detailed balance in equation 23. Overall, we used equation 23 for its preservation of the stationary distribution and its ease of implementation.

As stated earlier, imposing detailed balance on a fit to traces from molecules in thermal equilibrium will not hurt the quality of the fit much, since the inferred A almost satisfies detailed balance. On the other hand, imposing this condition on a fit to traces from molecules far from thermal equilibrium will result in A_{db} differing substantially from A and thus hurting the fit more. We can thus detect systems far from thermal equilibrium by trying both the constrained and unconstrained detailed balance fit and comparing the resulting $\log p(\text{data}\ |\theta_{\text{MLE}})$. To see if the resulting reduction in data likelihood justifies imposing detailed balance we again turn to the BIC. The number of free parameters eliminated by imposing detailed balance for a K state Markov model with P forbidden pairs of transitions is

$$\begin{aligned} f_{\text{elim,db}} = \ & [\text{number of edges in complete graph on states}] \\ & - [\text{number of edges in spanning tree on states}] - P \\ = \ & K(K-1)/2 - P - (K-1) = (K^2 - 3K)/2 + 1 - P \end{aligned} \tag{24}$$

We can now apply equation 8 with $d \to d - f_{\text{elim,db}}$ to compute the BIC for the constrained and unconstrained detailed balance model. Note that setting just $a_{i,j}$ equal to 0, but not $a_{j,i}$, is in general a constraint that may not be compatible with detailed balance, so we must set $a_{j,i} = 0$ as well to enforce $a_{\text{db},\ i,j}$.

Continuous to discrete time models

We have so far discussed only HMM's that produce emissions and switch states at discrete series of times. In studying single-molecule signals we are, however, often working with systems that evolve continuously, but which are sampled at some finite rate $f_\mathrm{s} = 1/\Delta t_\mathrm{s}$, where Δt_s is the time between successive samples. It is not in general correct to obtain continuous-time frequencies by multiplying the transition probabilities by a factor of f_s, since as probabilities the elements of the transition matrix A are bounded from above by 1, whereas frequencies are not. This approximation holds so long as the entries $a_{i,j}$ are small for each pair of states (i, j). In a continuous-time setting, the elements of A take on the interpretation

$$a_{ij} = \mathrm{P}(x_{t+\Delta t_\mathrm{s}} = j | x_t = i) \tag{25}$$

For any sampling frequency, there is some probability that multiple hops occur during a single sampling period Δt_s. Since a discrete-time approximation of the system only considers the endpoints of each sampling period, the quantity $a_{ij} f_\mathrm{s}$ is an underestimate of the true continuous-time hopping rate between the states, and a gross underestimate for sampling rates comparable to the inferred hopping rates. We can convert discrete to continuous rates via the relation

$$F = I + f_\mathrm{s} \cdot \log A \tag{26}$$

where log denotes a matrix (not element-wise) logarithm of A and I is the identity matrix. The elements of $(F)_{ij} = f_{ij}$ are the frequencies for transitions from state i to state j in units of s^{-1}. For the case of a two-state system, equation 26 implies that we need to rescale both hopping frequencies by the same factor

$$f_{12} = \frac{-\log(1 - a_{12} - a_{21})}{a_{12} + a_{21}} \cdot f_s \cdot a_{12} \tag{27}$$

For small values of $a_{12} + a_{21}$, the numerical correction pre-factor is approximately $1 + (a_{12} + a_{21})/2$.

References

[1] Kienker P (1989) Equivalence of aggregated Markov-models of ion-channel gating. Proc Royal Soc London Ser B-Biol 236: 169-309.

[2] Giudici P VP Ryden T (2000) Likelihood-ratio tests for hidden Markov models. Biometrics 56: 742-747.

[3] Bickel PJ RT Ritov Y (1998) Asymptotic normality of the maximum-likelihood estimator for general hidden Markov models. Annals of Statistics 26: 1614-1635.
